# Kinin B1 receptor mediates acute cardiovascular and neural responses following cannabinoid receptor 1 activation in conscious male mice

**DOI:** 10.14814/phy2.70579

**Published:** 2025-09-21

**Authors:** Drew Theobald, Syed Anees Ahmed, Abdel A. Abdel‐Rahman, Srinivas Sriramula

**Affiliations:** ^1^ Department of Pharmacology and Toxicology Brody School of Medicine at East Carolina University Greenville North Carolina USA

**Keywords:** blood pressure regulation, cannabinoid receptor 1, kinin B1 receptor, mitochondrial dysfunction, oxidative stress

## Abstract

The cannabinoid receptor 1 (CB1R) regulates cardiovascular functions and is activated by agonists such as WIN55,212‐2. While CB1R activation is known to influence blood pressure and oxidative stress both centrally and peripherally, the downstream mechanisms remain unclear. The kinin B1 receptor (B1R), induced by stress and inflammation, may function as a signaling partner for CB1R. This study investigated whether CB1R‐mediated effects require B1R activation. Wild‐type (WT) and B1R knockout (B1RKO) mice were treated with WIN55,212‐2 acutely. In WT mice, WIN55,212‐2 increased blood pressure, CB1R and B1R expression, and oxidative stress in the brain and heart, while these effects were absent in B1RKO mice. In vitro, H9c2 cardiomyocytes, human cardiac fibroblasts, and primary neurons were treated with WIN55,212‐2 with or without a B1R antagonist. WIN55,212‐2 increased CB1R and B1R expressions, and oxidative stress in all cell types and reduced mitochondrial membrane potential in H9c2 cells. In neurons, WIN55,212‐2‐induced mitochondrial and oxidative stress responses were attenuated by B1R inhibition. These findings reveal that CB1R activation acutely engages B1R signaling to drive pressor responses, oxidative stress, and mitochondrial dysfunction, positioning B1R as a critical downstream effector of CB1R. Targeting B1R may represent a novel strategy to mitigate cannabinoid‐induced oxidative stress and cardiovascular effects.

## Graphical abstract

Proposed role of kinin B1 receptor in cannabinoid receptor 1 mediated regulation of blood pressure.

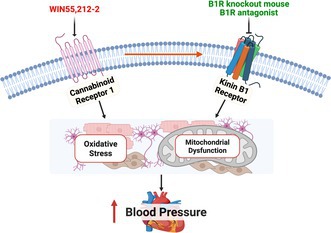

## 1 INTRODUCTION

Cannabinoid receptor 1 (CB1R), a G protein‐coupled receptor (GPCR), is widely expressed in the central nervous system (CNS) and peripheral organs and has been found to be upregulated in various cardiovascular disease states and may influence disease progression (Alfulaij et al., 2018). CB1R activation induces different cardiovascular responses that are dependent on whether it is administered centrally or systemically and whether the study is conducted on anesthetized or conscious animals (Ibrahim & Abdel‐Rahman, 2014). Previous studies have shown that systemic administration of WIN55,212‐2, a synthetic CB1R ligand, in conscious animals predominantly elicits sympathoexcitation and pressor responses (Ibrahim & Abdel‐Rahman, 2012a, 2015). In contrast, cannabinoids administered systemically in anesthetized animals resulted in bradycardia and hypotension (Lake et al., 1997; Zakrzeska et al., 2010). In the CNS, cardiovascular regulatory nuclei such as the nucleus tractus solitarius (NTS) and the rostral ventrolateral medullar (RVLM) have been implicated in neuroinflammatory tone and autonomic outflow induced by central CB1R activation (Ibrahim & Abdel‐Rahman, 2011, 2012b; Pfitzer et al., 2004; Rademacher et al., 2003; Zakrzeska et al., 2010). In the heart, CB1R activation by synthetic ligands or endocannabinoids promotes cardiac dysfunction by increasing inflammation and oxidative stress in endothelial cells, cardiomyocytes, and inflammatory cells (Han et al., 2009; Mukhopadhyay et al., 2010; Rajesh et al., 2010).

Recent studies have suggested that GPCRs can engage in receptor–receptor interactions, including crosstalk and transactivation, which may modulate CB1R effects (Haspula & Clark, 2020; Ibrahim & Abdel‐Rahman, 2015; Mińczuk et al., 2022). The kinin B1 receptor (B1R) plays a pivotal role in inflammation and oxidative stress, especially within the cardiovascular system and CNS. B1R is an inducible GPCR that is present at low levels during physiological conditions but is upregulated in response to inflammation or injury (Sriramula, 2020; Theobald & Sriramula, 2023). Previous studies have shown that B1R upregulation can exacerbate oxidative stress and promote inflammation, suggesting it may serve as a mediator of pathological signaling cascades associated with CB1R activation (Dias et al., 2015; Pouliot et al., 2012; Theobald & Sriramula, 2023).

It has recently been suggested that CB1R and B1R engage in functional crosstalk (Lim & Park, 2012; Pelorosso et al., 2009). Both receptors can signal through Gαi/o and Gαq proteins to modulate MAPK signaling and intracellular calcium. Given their similar localizations in the CNS and cardiovascular systems and shared downstream targets, CB1R activation may induce or require B1R signaling through transactivation or bidirectional crosstalk. However, the directionality and underlying mechanisms of this interaction in cardiovascular regulation during stress remain largely unknown. Additionally, whether these interactions are conserved or differ across organ systems remains unexplored.

In the current study, we investigated the acute effects of CB1R activation on pressor response and oxidative stress within the brain and heart and determined if B1R is required for the aforementioned effects. WIN55,212‐2 was blindly administered systemically via the jugular vein in conscious wild‐type (WT) and B1R knockout (B1RKO) mice. This study focused on the paraventricular nucleus (PVN) of the hypothalamus, a key brain region involved in autonomic and cardiovascular regulation, as well as the heart, where both cannabinoid and kinin systems play critical roles. We further used primary neuronal, cardiac fibroblast, and cardiomyocyte cultures to dissect cellular mechanisms and explore potential CB1R–B1R crosstalk in vitro.

## 2 MATERIALS AND METHODS

### 2.1 Animals

Male mice were housed in a humidity‐ and temperature‐controlled facility (23 ± 1°C) under a 12‐h dark/light cycle. Mice were given water ad libitum and fed standard mouse chow, Prolab IsoPro RMH 3000, #3005737‐220, Lab Diet. Bradykinin B1 receptor knockout (B1RKO) mice were a generous gift from Dr. Michael Bader (Charité Hospital, Berlin, Germany) and originated from the backcrossing of an initially mixed genetic background (129/Sc and C57Bl/6) with C57Bl/6 mice. Wild‐type (WT) C57Bl/6NJ mice (stock no. 005304) were purchased from the Jackson Laboratory. Only male mice were used to minimize variability associated with the estrous cycle, which previous studies have shown can affect endocannabinoid signaling in cardiovascular and inflammatory pathologies (Forner‐Piquer et al., 2024; Riebe et al., 2010). All animal studies were approved by the East Carolina University Animal Care and Use Committee (AUP #W261a) and were performed in accordance with the National Institutes of Health (NIH) Guidelines for the Care and Use of Laboratory Animals and ARRIVE guidelines.

### 2.2 Blood pressure measurements

The synthetic cannabinoid receptor agonist WIN 55,212‐2 was used to evaluate CB1R‐mediated blood pressure responses in WT and B1RKO mice. This model system enables comparison of the CB1R‐induced pressor response and the role of B1R in modulating these responses. Direct arterial blood pressure recordings were obtained in WT and B1RKO mice to evaluate the acute hemodynamic effects of WIN 55,212‐2. Mice were acclimated for at least 30 min before anesthesia induction using intraperitoneal ketamine (90 mg/kg) and xylazine (10 mg/kg). Anesthesia depth was confirmed by the absence of the pedal withdrawal reflex. The right carotid artery and jugular vein were catheterized under aseptic conditions using a MAC‐2B catheter filled with heparinized saline (100 IU/mL). Catheters were connected to a pressure transducer interfaced with a PowerLab 8/30 data acquisition system (ADInstruments). Arterial pressure was continuously recorded using LabChart Pro software, and mean arterial pressure (MAP) was calculated. After a stable 30‐min baseline, WT and B1RKO mice were randomized into groups, and WIN 55,212‐2 (400 μg/kg, W102 Sigma) or vehicle (saline) was administered intravenously. MAP was monitored for 45 min in conscious mice, and data were expressed as changes in MAP relative to baseline. All procedures complied with institutional animal care guidelines, and all experiments and analysis were performed in a blinded fashion by two independent investigators.

### 2.3 Primary neuron culture

Primary neurons were cultured from WT neonatal mice pups as previously described (Parekh et al., 2020, 2021; Theobald & Sriramula, 2023; White et al., 2022). Mouse pups were anesthetized with isoflurane (4%) in an oxygen flow (1 L/min) before decapitation, and brains were collected, and the hypothalamus was retrieved. The tissue was digested with HBSS containing 1% trypsin (T1426 Sigma‐Aldrich, St. Louis, MO, United States) and 1.5 kU/mL DNaseI (D5025 Sigma‐Aldrich) for 10 min at 37°C. Cells underwent centrifugation and were then resuspended in complete Neurobasal culture medium (21103049 Gibco) supplemented with 2% B27, 0.5 mM GlutaMax, and penicillin/streptomycin (100 U/mL and 100 μg/mL, respectively) (Gibco). Neurons were then plated into poly‐L‐lysine coated cell culture plates and grown in a humidified atmosphere of 5% CO_2_–95% air at 37°C and treated with cytosine arabinofuranoside (Ara‐C, 2 μM, C1768 Sigma‐Aldrich) to arrest the growth of non‐neuronal cells. Neurons were cultured for at least 10 days prior to experimentation. The cells were pretreated with R715 (3407, Tocris Bioscience, 10 μM) or vehicle 2 h prior to the addition of WIN55,212‐2 (W102 Sigma‐Aldrich, 1 μM) or vehicle (sterile PBS) for 24 h. The treatment durations and doses were based on our preliminary studies and published literature (Lott et al., 2022; Parekh et al., 2020; Pérez‐Diego et al., 2023; Theobald & Sriramula, 2023; Xu et al., 2015).

### 2.4 H9c2 cell culture

H9c2 cells were purchased from ATCC (CRL‐1446) and grown following manufacturers' recommendations. Cell pellet was resuspended in complete medium (Dulbecco's Modified Eagle's Medium supplemented with 10% fetal bovine serum) and grown in a humidified atmosphere of 5% CO_2_–95% air at 37°C. The cultures were passaged after 70%–80% confluence was achieved. Cells were rinsed with a PBS solution, then 3 mL of trypsin–EDTA solution was added to the flask for 5 min. Cells were spun down, and 7 mL of complete growth medium was added, and cells were plated in new culture vessels at a ratio of 1:3. Medium was changed every 2–3 days. The cells were pretreated with R715 (#3407, Tocris Bioscience, 10 μM) for 2 h prior to the addition of WIN55,212‐2 (W102 Sigma‐Aldrich, 1 μM) or vehicle (sterile PBS) for 24 h.

### 2.5 Human cardiac fibroblast culture

Human cardiac fibroblasts obtained from the ventricles of a 15‐year‐old male were purchased from PromoCell (C‐12375) and cultured per the manufacturer's protocol. Cells were cultured using Fibroblast Growth Medium 3 (Promo Cell C‐23025) in T‐75 cell culture flasks and grown in a humidified atmosphere of 5% CO₂ and 95% air at 37°C. Cells were passaged using PromoCell DetachKit (C‐41200) after reaching 80% confluency by rinsing with HEPES prior to the addition of Trypsin/EDTA for 5 min. Then, cells were incubated with trypsin neutralizing solution prior to centrifugation. Cell pellets were resuspended in media and plated in new cell culture vessels. Prior to treatment, cells were switched to low‐serum media (PromoCell C‐23020). Cells were pretreated with R715 (#3407, Tocris Bioscience, 10 μM) for 2 h prior to the addition of WIN55,212‐2 (W102 Sigma‐Aldrich, 1 μM) or vehicle (sterile PBS) for 24 h.

### 2.6 Immunofluorescence staining

Cells and tissue were fixed with 4% paraformaldehyde for 15 min. Samples were washed 3 times with 1×PBS and blocked with 2% normal donkey serum (017‐000‐121, Jackson ImmunoResearch), 0.05% Tween 20 (P9416, Sigma), 50 mM glycine (G8898, Sigma), 0.1% Triton‐X 100 (X100, Sigma), and 0.01% BSA (A9647, Sigma) in 1×PBS for 60 min at room temperature. Samples were incubated at 4°C overnight in B1R (1:250, ABR‐011, Alomone Labs), CB1R (1:200, custom), or MAP2 (1:500, NBP3‐05552, Novus Biologicals). The following day, samples were washed three times for 10 min in 0.5% Tween 20 in 1×PBS. Immunolabeling was done with appropriate Alexa Fluor 488 (Donkey anti‐mouse Alexa Fluor Plus 488, A32766 Invitrogen, 1:1000 dilution), 555 (Donkey anti‐Rabbit Alexa Fluor Plus 555, A32794 Invitrogen, 1:1000 dilution), and 647 (Donkey anti‐goat Alexa Fluor Plus 647, A32849 Invitrogen, 1:1000 dilution) conjugated secondary antibodies for 2 h at room temperature. Sections were mounted with Vectashield Vibrance Antifade Mounting Medium with DAPI (H‐1800, Vector Laboratories), and images were captured using a fluorescence microscope with 20× and 40× objective lenses (Keyence/Echo revolve). Mean fluorescent intensity was quantified using ImageJ software (NIH) and presented in graphs as fold change of immunofluorescence staining (% area) relative to vehicle treated. All experiments and analyses were performed in a blind fashion by two independent investigators.

### 2.7 Measurement of ROS levels

Reactive oxygen species levels were measured using dihydroethidium (DHE) staining (D11347, ThermoFisher). The heart and brain were embedded in tissue freezing medium (O.C.T. Compound, Fisher 4585) and cut into 10 μm sections using a cryostat. Sections or cells were treated with 10 μM DHE and incubated in a light‐protected humidified chamber at 37°C for 15 min. Fluorescence was detected at excitation/emission 518/605 nm.

### 2.8 Mitochondrial dysfunction measurements

Mitochondrial superoxide levels were assessed using the MitoSOX Red mitochondrial superoxide indicator (Invitrogen, M36008). A 5 μM working solution was prepared by diluting the 5 mM MitoSOX stock solution in pre‐warmed Hanks' Balanced Salt Solution (HBSS). Cell culture media were removed from each well, and MitoSOX solution was added gently to each sample, then incubated at 37°C for 10 min, protected from light. After incubation, cells were washed three times with pre‐warmed HBSS to remove excess dye, and fluorescence was measured using a spectrofluorometer where fluorescence was detected at excitation/emission wavelengths of 510/580 nm.

Mitochondrial membrane potential was measured using tetramethylrhodamine ethyl ester perchlorate (TMRE, Invitrogen Cat# T669). A TMRE working solution was prepared fresh by diluting the manufacturer's stock solution (1 mM in DMSO) into pre‐warmed, serum‐free medium. Cells were incubated with TMRE at 37°C for 30 min in the dark at a final concentration of 100 nM. Following incubation, the dye‐containing medium was gently removed, and cells were washed twice with warm HBSS to remove excess dye. Fluorescence was detected at excitation/emission wavelengths of 549/574 nm.

### 2.9 Statistics

Data are presented as mean ± SD. Statistical analyses were performed using GraphPad Prism 10.0.1 (GraphPad Software). Multiple comparisons were made using one‐way ANOVA or two‐way ANOVA, followed by Tukey's multiple comparisons test, as appropriate. Differences were considered statistically significant at *p* < 0.05, and specific significant *p* values are given on the graphs. For cell culture experiments, results were analyzed from at least three independent cultures. For in vivo studies, the number of animals per group is indicated in the figure legends, and a power analysis was conducted during study design to ensure adequate sample size.

## 3 RESULTS

### 3.1 WIN55,212‐2 induces a pressor response in WT but not in B1RKO mice

**FIGURE 1:**
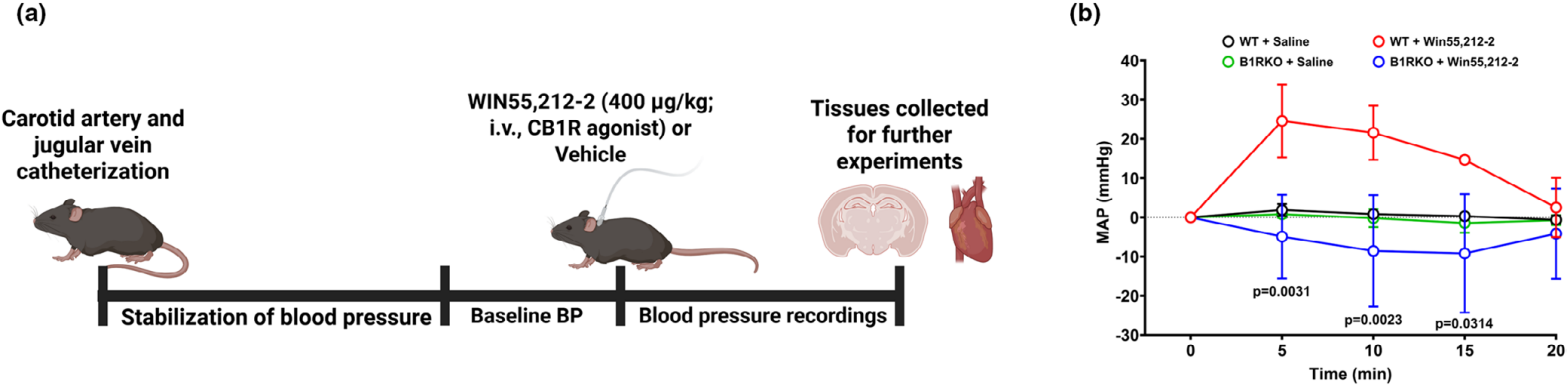
WIN55,212‐2 increases blood pressure in a B1R‐dependent manner. (a) Experimental schematic showing acute intravenous injection of WIN55,212‐2 (400 μg/kg) via the jugular vein, followed by blood pressure measurement and tissue collection. (b) Mean arterial pressure (MAP) response to WIN55,212‐2 in wild‐type (WT) and B1 receptor knockout (B1RKO) mice. WIN55,212‐2 significantly increased MAP in WT but not in B1RKO mice, suggesting a B1R‐dependent mechanism. Data are presented as mean ± SD. Repeated measures 2‐way ANOVA, *n* = 3–6 mice/group.

Following a stabilization period of at least 30 min, conscious mice received either CB1R agonist WIN55,212‐2 (400 μg/kg) or vehicle (saline), and blood pressure recordings continued for 45 min (Figure 1a). At baseline, there were no differences in mean arterial pressure values across groups (WT: 86.26 ± 47.05 mmHg; B1RKO: 78.90 ± 4.88; *p* = 0.788). WIN55,212‐2 significantly (*p* < 0.05) elevated mean arterial pressure (ΔMAP) in WT mice, but this effect was substantially attenuated (*p* < 0.05) in B1RKO mice (Figure 1b).

### 3.2 CB1R activation increases oxidative stress through B1R

**FIGURE 2:**
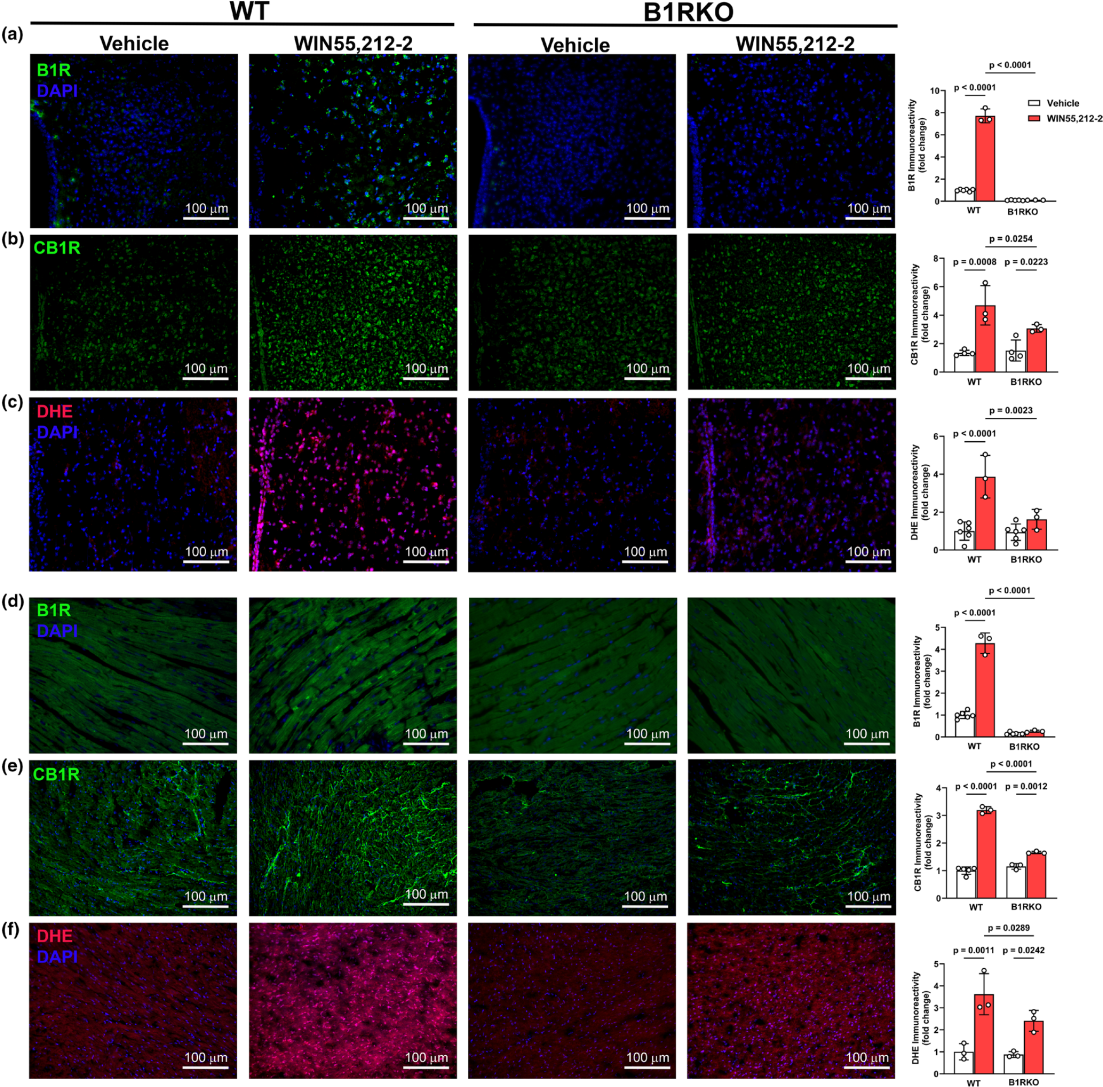
Brain and cardiac expression of B1R, CB1R, and oxidative stress following WIN55,212‐2 administration. Representative images and quantification of B1R (a), CB1R (b), and oxidative stress (DHE, c) expression in the paraventricular nucleus (PVN) of the hypothalamus. Images and corresponding expressions of B1R (d), CB1R (e), and oxidative stress (DHE, f) in heart tissue. WIN55,212‐2 increased CB1R expression and oxidative stress in WT but not B1RKO mice in both brain and heart. Data are presented as mean ± SD. *n* = 3–6 mice/group; 2‐way ANOVA with Tukey's multiple comparisons test.

Compared to vehicle, WIN55,212‐2 (*p* < 0.05) increased B1R (Figure 2a) and CB1R (Figure 2b) expressions in the PVN of WT mice but had minimal effects on their expressions in B1RKO mice. Similarly, WIN,55212‐2 (*p* < 0.05) increased DHE fluorescence (Figure 2c), indicative of increased oxidative stress in the PVN of WT mice, but not in B1RKO mice. Similar results were observed in the heart, where WIN55,212‐2 increased (*p* < 0.05) B1R (Figure 2d) and CB1R (Figure 2e) expressions and DHE staining (Figure 2f) in WT mice, but not in B1RKO mice. These findings support a role for B1R in CB1R‐mediated redox stress in both the brain and heart.

### 3.3 Differential CB1R‐B1R crosstalk in cardiac fibroblasts and cardiomyocytes

**FIGURE 3:**
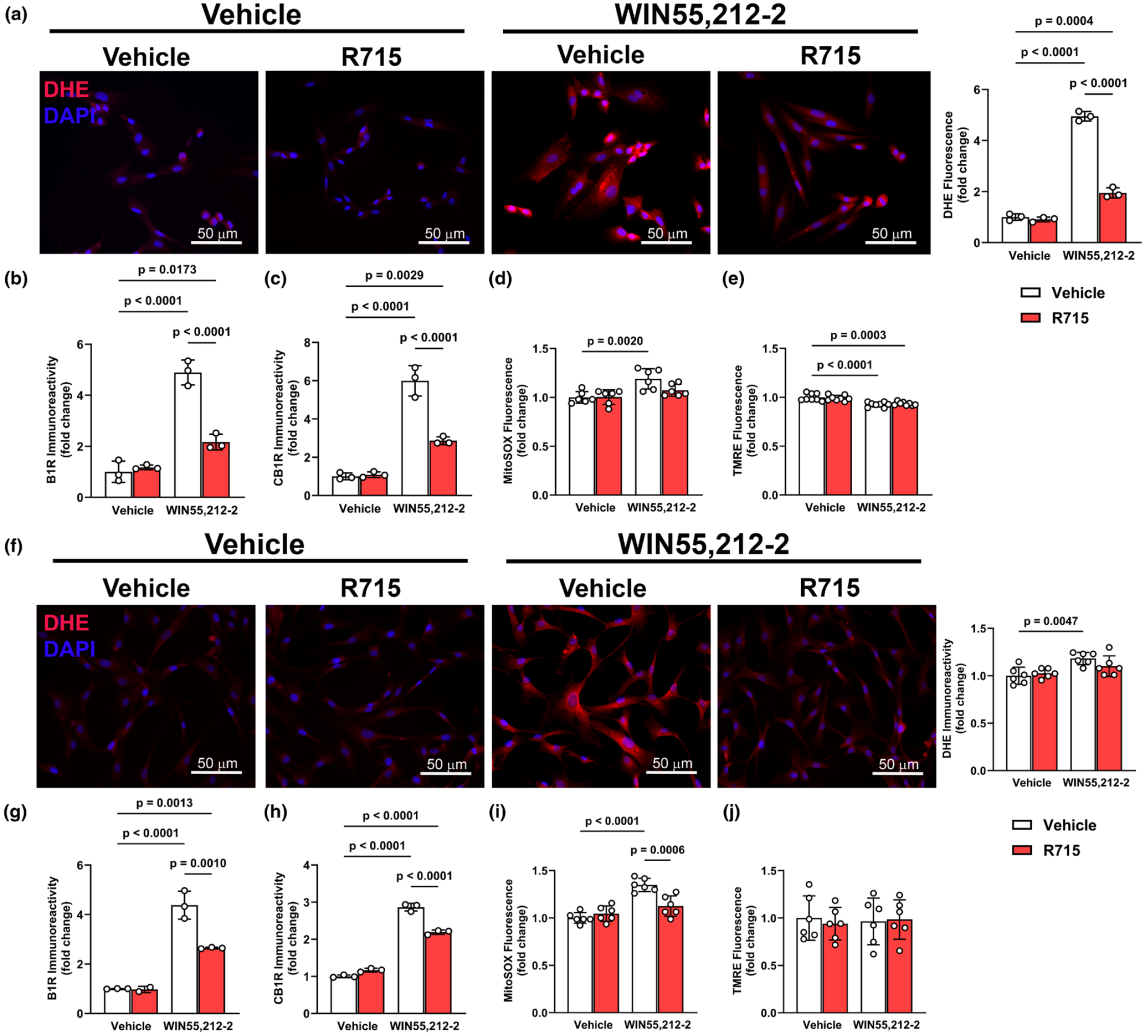
Effects of WIN55,212‐2 on oxidative stress and mitochondrial function in H9c2 cardiomyocytes and human cardiac fibroblasts. Quantification of DHE (a), B1R (b), CB1R (c), mitochondrial superoxide (MitoSOX, d), and mitochondrial membrane potential (TMRE, e) in H9c2 cardiomyocytes following WIN55,212‐2 treatment. WIN55,212‐2 in H9c2 cells increased oxidative stress, B1R, and CB1R expression, and decreased mitochondrial membrane potential. Corresponding quantification of DHE (f), B1R (g), CB1R (h), MitoSOX (i), and TMRE (j) in human cardiac fibroblasts. Unlike H9c2 cells, WIN55,212‐2 did not significantly alter TMRE in HCFs, despite increasing oxidative stress and receptor expression. Data are presented as mean ± SD. *n* = 3–6 independent cultures/group: One‐way ANOVA followed by Tukey's multiple comparisons.

To assess whether CB1R‐B1R interactions regulate oxidative and mitochondrial stress in cardiac cell types, we treated H9c2 cardiomyocytes and human cardiac fibroblasts (HCFs) with WIN55,212‐2, with or without the B1R selective antagonist, R715. In H9c2 cells, WIN55,212‐2 (*p* < 0.05) increased DHE fluorescence (Figure 3a), indicating elevated cytosolic ROS. This was accompanied by upregulations (*p* < 0.05) of both B1R and CB1R (Figure 3b,c). Mitochondrial‐specific ROS, measured by MitoSOX, was also increased (*p* < 0.05) following WIN55,212‐2 exposure (Figure 3d), and this effect was attenuated by B1R blockade, suggesting that B1R contributes to WIN55,212‐2‐induced mitochondrial oxidative stress. However, in H9c2 cells, WIN55,212‐2 significantly (*p* < 0.05) reduced mitochondrial membrane potential as measured by TMRE (Figure 3e), indicating early mitochondrial dysfunction, and this effect was not prevented by B1R blockade. In HCFs, a similar increase in DHE, B1R, and CB1R was observed following WIN55,212‐2 treatment (Figure 3f–h), along with increased MitoSOX signal (Figure 3i) that was again blunted by B1R blockade. However, in contrast to H9c2 cells, TMRE fluorescence remained unchanged in HCFs following exposure to WIN55,212‐2 (Figure 3j), indicating preserved mitochondrial membrane potential.

### 3.4 Dependence of CB1R‐induced ROS and inflammation on B1R in primary neurons

**FIGURE 4:**
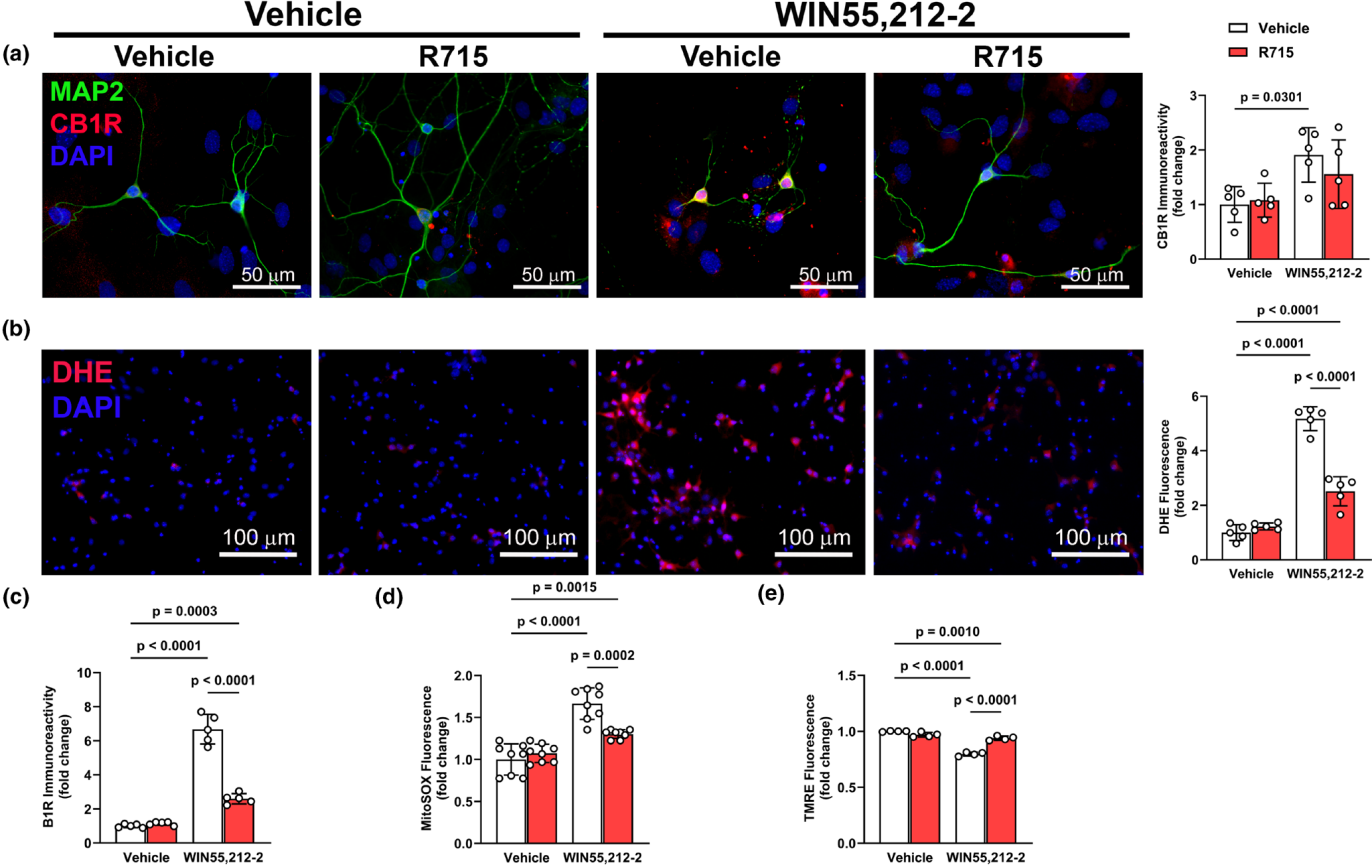
WIN55,212‐2‐induced effects in primary neurons are mediated in part by B1R. Quantification of CB1R (a), DHE (b), and B1R (c) expression following WIN55,212‐2 with or without B1R antagonist pretreatment. WIN55,212‐2 increased CB1R, B1R and oxidative stress, and was attenuated by pharmacological inhibition of B1R. Mitochondrial superoxide (MitoSOX, d) and membrane potential (TMRE, e) showed impaired mitochondrial function with WIN55,212‐2, partially rescued by B1R antagonism. Data are presented as mean ± SD. *n* = 5–8 independent cultures/group: One‐way ANOVA followed by Tukey's multiple comparisons.

In cultured primary neurons, WIN55,212‐2 increased (*p* < 0.05) CB1R (Figure 4a) and B1R (Figure 4b) expressions along with increasing (*p* < 0.05) DHE fluorescence (Figure 4c). Co‐exposure to a B1R antagonist, R715, significantly (*p* < 0.05) attenuated the effects of WIN55,212‐2, indicating that B1R contributes to CB1R‐mediated oxidative stress. Consistent with the idea that CB1R‐B1R interactions impair mitochondrial function, WIN55,212‐2 (*p* < 0.05) increased MitoSOX signal (Figure 4d) and reduced TMRE fluorescence (Figure 4e) in primary neurons. Notably, B1R blockade mitigated all WIN55,212‐2‐induced effects, linking B1R signaling to CB1R‐driven mitochondrial stress in neurons and a potential transactivation or crosstalk between CB1R and B1R in regulating mitochondrial homeostasis.

## 4 DISCUSSION

This study showed that in conscious mice, systemic administration of the cannabinoid receptor agonist WIN55,212‐2 induces a pressor response along with neuronal (PVN) and cardiac oxidative stress, at least partly, via kinin B1R. Moreover, the in vitro studies confirmed a pivotal role for kinin B1R in CB1R‐induced mitochondrial oxidative stress across multiple cell types, except for a cell‐specific effect on mitochondrial membrane potential. Collectively, this study identifies a functional and potentially targetable interaction between CB1R and B1R, significantly advancing our understanding of the molecular pathways that underlie acute cannabinoid‐induced cardiovascular and neuroinflammatory responses.

While earlier studies reported CB1R‐mediated hypotensive effects likely due to the use of anesthetized animals, more recent research supported a centrally mediated pressor response in conscious animals (Benyó et al., 2016; Ibrahim & Abdel‐Rahman, 2012a, 2015; Wheal et al., 2007). Our novel finding that WIN55,212‐2 induces an acute increase in blood pressure in conscious WT, but not in B1RKO mice, underscores the importance of the B1R in this CB1R‐mediated effect. This postulate builds upon previous studies showing that B1R is upregulated in the PVN in response to hypertensive stimuli (Parekh et al., 2021; Sriramula & Lazartigues, 2017), where it contributes to oxidative stress and impaired autonomic function. Our data suggest that CB1R may act upstream of, or in a feedforward manner with, B1R to elicit similar outcomes.

Although functional interactions between CB1R and other GPCRs such as AT1R, D2R, and OX1R receptors have been described (Ibrahim & Abdel‐Rahman, 2015; Mińczuk et al., 2022; Muñoz‐Arenas et al., 2015; Rivas‐Santisteban et al., 2024; Rozenfeld et al., 2011), very few studies have investigated CB1R interactions with B1R. Our data showed that CB1R activation increased B1R expression and likely engaged B1R‐dependent signaling in the molecular responses including enhanced oxidative stress and the increase in blood pressure in WT mice. While the rapid increase in B1R expression and signaling was somewhat surprising, these findings are consistent with studies that reported heterologous sensitization between GPCRs or shared intracellular effectors such as β‐arrestins (Carmona‐Rosas et al., 2019; Rajagopal & Shenoy, 2018; Smith & Rajagopal, 2016). Additionally, studies in epithelial and colon cells have shown that CB1R activation can transactivate EGFR or induce redox signaling cascades that converge on inflammatory pathways (Deng et al., 2022; Haspula & Clark, 2020; Yang et al., 2010), suggesting that receptor crosstalk may underlie diverse effects of cannabinoids in different systems. Nonetheless, it was important to confirm a causal role for B1R in CB1R‐mediated effects in WT mice in our study. This goal was achieved by demonstrating convincing evidence by the lack of the CB1R‐induced: (1) oxidative stress and pressor response in B1RKO mice in vivo, and (2) molecular effects in cultured cells in vitro.

Our in vitro results demonstrate cell‐specific mitochondrial responses to CB1R activation and their dependence on B1R. Both H9c2 cardiomyocytes and human cardiac fibroblasts exhibited WIN55,212‐2‐induced oxidative stress, which was prevented by B1R blockade. However, only cardiomyocytes displayed a significant decrease in mitochondrial membrane potential, and this effect was not prevented by B1R blockade. These findings suggest that while CB1R–B1R interaction is critical for ROS production, mitochondrial depolarization may occur through a B1R‐independent pathway in some tissues.

Interestingly, previous studies in the CNS have shown CB1R to be localized to the mitochondria in neurons, where it regulates energy metabolism and ROS generation (Hebert‐Chatelain et al., 2016). In our primary neurons, WIN55,212‐2 increased CB1R and B1R expression, mitochondrial oxidative stress, and mitochondrial membrane depolarization. However, B1R blockade attenuated oxidative stress, but not membrane potential loss. These findings corroborate the idea that CB1R‐B1R crosstalk selectively modulates redox signaling, while mitochondrial bioenergetic dysfunction may be a parallel or downstream consequence of CB1R activity.

A major contribution of this study is the inclusion of both brain and heart tissues, as well as three distinct cell types (neurons, cardiomyocytes, and fibroblasts). This approach revealed context‐dependent outcomes of CB1R‐B1R interactions. In the CNS, the CB1R‐B1R axis drove oxidative stress and mitochondrial stress, consistent with the PVN's role in integrating autonomic and neuroimmune signals (Ferguson et al., 2008). In the heart, both contractile (H9c2) and noncontractile (HCF) cells responded to CB1R activation with B1R‐dependent oxidative stress. However, only cardiomyocytes exhibited mitochondrial depolarization, likely due to their high energy demand and mitochondrial content (Brown et al., 2017).

This study focused solely on male mice, leaving open the question of whether CB1R‐B1R interactions are sex dependent. Given well‐documented sex differences in cardiovascular regulation, immune responses, and endocannabinoid system function, future studies should investigate sex as a biological variable in this area of research. Along with this, the small number of animals used for in vivo experiments were small and future studies should increase the number of mice; however, the reproducibility of these findings across multiple cell models provides strong complementary support for the observed mechanisms. Additionally, while our experimental design leveraged multiple models that strengthen translational relevance, it also introduces potential inconsistencies. Species‐specific differences in receptor expression and intracellular signaling may affect the responses to CB1R activation and B1R modulation. While unlikely given the expression of both receptors across species, studies are still needed to determine if the magnitude of the interaction is species specific or is altered in coculture systems. In addition, we did not measure arterial blood gases, which restricts our ability to fully evaluate the contribution of respiratory or metabolic changes to the observed outcomes. Although CB1R expression is known to vary with circadian rhythm, we did not examine the circadian rhythm effects. It is notable, however, that all experiments were conducted at the same time of day and under the same conditions to minimize variability related to circadian rhythm. Future studies will determine if the circadian rhythm influences CB1R‐mediated effects in our model system. Furthermore, while we demonstrated receptor expressions and functional dependence, we did not directly assess whether CB1R and B1R physically interact or form heterodimers. Another limitation is small sample size and the acute administration of WIN55,212‐2 and limiting its effects to 30 min, which also resulted in appreciable variability in heart rate responses. Interestingly, however, the findings might be clinically relevant because studies revealed serious cardiac effects within 60 min of Δ^9^‐THC administration (Pabon et al., 2022), and WIN55,212‐2 and Δ^9^‐THC exhibit similar cannabinoid receptors agonist profiles (Laaris et al., 2010). Nonetheless, it remains unknown if similar CB1R‐B1R crosstalk persists or adapts during chronic exposure, which is more relevant to therapeutic or recreational cannabinoid use, and deserves further investigation.

In conclusion, this study reveals a novel and functionally significant role for B1R in the acute CB1R‐mediated oxidative stress and blood pressure response. By combining in vivo and in vitro approaches, we demonstrate that B1R plays a necessary role in mediating the pressor response and downstream oxidative and mitochondrial stress triggered by CB1R activation. These findings not only identified noncanonical CB1R signaling but also highlight B1R as a potential therapeutic target to mitigate oxidative stress related to hyperactive endogenous CB1R signaling.

## AUTHOR CONTRIBUTIONS

S.S. and A.R. conceptualized and designed research; D.T. and S.S. designed experiments, performed experiments, analyzed data, wrote the initial paper, and revised the paper. S.A. performed some experiments and revised the paper. S.S. and A.R. edited the final manuscript. All authors have read and agreed to the published version of the manuscript.

## FUNDING INFORMATION

This study was supported by the National Heart, Lung, and Blood Institute of the National Institute of Health under award number 5R01HL153115 (Dr. Sriramula) and by the National Institute on Alcohol Abuse and Alcoholism grant R01 AA14441‐15 (Dr. Abdel‐Rahman).

## CONFLICT OF INTEREST STATEMENT

The authors have declared that no conflict of interest exists.

## ETHICS STATEMENT

All animal protocols in the present study were approved by the Brody School of Medicine at East Carolina University’s IACUC.

## Data Availability

The authors declare that all the data supporting the findings of this study are contained within the paper.
